# Annotations

**Published:** 1899-04-15

**Authors:** 


### Annotations.

### The Ownership of Hospital Case-Books.

What to do with the case-books which a physician accumulates as years go by seems sometimes to be a difficult matter to decide. In our opinion, even where there is a partner in the concern, it is a very serious ethical question whether the personal note-books of a deceased physician should be handed over to him, but in all other cases there can be no doubt that they should be at once destroyed by the executors. A curious little scandal in connexion with the Hospital for Women in Soho Square has been brought to light by a paragraph in *Truth*, describing how at a recent auction at Messrs. Puttick and Simpson's a lot was sold containing among other things 21 old manuscript volumes of cases treated at that hospital by the late Dr. Alfred Meadows! A gentleman who happened to see the books, and thought it an improper thing that such records should be offered for sale in a public auction-room, went round and remonstrated with the hospital authorities, who, however, it is stated, "ridiculed his views," whereupon he appealed to *Truth*. There can, we think, be no doubt about the gross impropriety of the proceeding in question, and, seeing that the whole lot only fetched 3s. 6d., we think that the hospital, after making the initial and inexcusable error of parting with its records, might well have made some effort to prevent them from falling into the hands of people who might put them to improper uses. It would be a matter of no small interest to know what is the ultimate fate of the case-books so carefully kept by some medical men. As a general thing, we are quite sure that case-taking is done in a very slipshod manner by general practitioners. But consulting men find it necessary to be able to lay their hands at once on the records of cases they may have seen, perhaps, only once, and that years ago, so that not infrequently they keep their books most systematically, even going so far sometimes as to have their notes typewritten, indexed, and bound up along with all the correspondence which has taken place with relatives and friends on the subject. Indeed, we can imagine that the case-books of some of our leading consultants, although overloaded, of course, with rubbish, might be really interesting "finds" for writers of biography. We hope, however, and we believe, that as a rule such papers are destroyed, although we confess that we do not see how anyone can be compelled to destroy them. But when we come to the question of hospital records the affair is quite different. They belong to the hospital, and the hospital does not die. If they are kept properly they should remain as a permanent record of the scientific work of the institution, and not be parted with on any consideration. It is not difficult to trace back the origin of this disagreeable incident to influences which we are afraid were common enough in the early history of many of the special hospitals which are scattered about London. We do but repeat what is perfectly well known when we say that most of these hospitals were founded at the instigation of medical men who were anxious to find a suitable field for the exercise and development of the specialties in which they were interested. Thus it happened that the personal element was always strong in the medical management of these concerns, and that things were done in them which no hospital of established position would ever have permitted. It is said that at this particular hospital it is the custom to present to each physician all the "letters" of his patients bound up at the end of every year. It need hardly be said how improper such a proceeding is, or how it takes away from the scientific value of the work done in the hospital. It is a boast and pride of our great hospitals that the work done in them is done openly, that it is recorded impartially, and that nothing can be hidden. But time after time events have shown that the smaller special hospitals have failed to follow in their footsteps. The records of cases attended at public hospitals are in no sense the property of the medical officers, but belong to the institutions; and if a hospital gives up possession of such records, even to the medical men who wrote them, it shows a complete misapprehension of the relations which ought to exist between the hospital, its medical staff, and its patients.

### The Medical Graduates' College and Polyclinic.

Although the formal opening of the Medical Graduates' College and Polyclinic will not take place until May, work will be commenced in the polyclinic next week, when the first of the consultations which are intended to be so prominent a feature of the clinical teaching at that institution will be held. In regard to the college we need not say more than that there is every reason to suppose that it will be of great direct utility to the medical profession, and indirectly of much advantage to the public. The polyclinic, however, must not be so ightly dismissed. Primarily, of course, its aim is educational, but so far as its patients are concerned it must take its place among the medical charities of the metropolis. No medicines will be given, no operations will be performed, no treatment will be undertaken, but each case will be thoroughly investigated, will be used as the subject of a clinical demonstration, and will receive through the medical man by whom it is sent up a carefully thought out opinion as to the nature of the disease and the best line of treatment to pursue. This will, we think, be a distinct novelty among the medical charities, and will meet a great want. There must be a very large number of patients who go to hospitals for the sake of what they call the "best advice," and who care nothing for the bottle of physic which accompanies it, and we feel sure that there are a very

ANNOTATIONS—*continued.*

large number of medical men who would be glad to have a second opinion on many of their poorer chronic and obscure cases if only the fee did not stand in the way; for although it is true that in many cases the fee difficulty can be overcome at the personal request of the doctor in attendance, there are not many medical men who would care often to place themselves under such obligations to their consultant friends; nor, indeed, has every man got consultant friends suited to all his cases. Now to these patients and to these medical men the polyclinic will be a great boon. If only the case is a reasonably typical one, or is obscure, or is moderately rare—if only it is something which can be made to serve as an illustration of a clinical demonstration—it will be gladly received, and the question of obligation between consultant and practitioner will not come in, while, so far as the patient is concerned, his security that he will get what he wants, namely, a complete investigation of his case and a careful opinion upon it, lies in the fact that everything will be done in public, and that the consultant's reward for what he does is the reputation as a "careful man," and as a "good opinion," which he earns among the practitioners who attend his class. The establishment of this institution is a most interesting experiment; and, although the "college" department should, of course, become self-supporting, there is much in these free consultations at the polyclinic which may well appeal for support to the charitable, promising, as they do, to offer to the poor some measure of that kind of help for which the rich have to pay in guineas.

### A Gross Fraud upon the Poor.

At an inquest recently held at Putney Mr. Braxton Hicks, the coroner, said he wished to take that opportunity of exposing what he characterised as a gross fraud upon the poor, and went on to describe how it had come out at an inquest upon a girl who had suffered from epilepsy that her father, a poor labouring man, seeing an advertisement headed, "I cure fits," had purchased a bottle of medicine from the advertiser, paying seven shillings for it. On analysis this medicine was found to contain three grains of bromide of potassium to the drachm, the whole bottleful costing about twopence. He could not help saying, as a coroner, that when he found poor people relying on that sort of thing and paying seven shillings a bottle for it, when sixpence would leave a very good margin for profit, he was bound to raise his voice and protest against it, for it was nothing less than a fraud upon the poor. With all of this we naturally agree, but we would point out that the iniquity laid bare by Mr. Braxton Hicks was mild indeed compared with what goes on every day. This man did at least put something into his bottle, and, notwithstanding the mendacity of the advertisement and the inefficiency of the dose, the drug which it contained did have some relation to the disease aimed at. This is much more than can be said for some much-advertised quack medicines. Twopence a bottle would, we fancy, go a long, long way in the purchase of the drugs used in the manufacture of some of them. But there is something much worse than mere monetary fraud about many quack medicines. To sell twopenny-worth of bromide to a poor man for seven shillings is bad enough, and Mr. Braxton Hicks has done well to lay bare the fraud, but we hope that he will find still stronger words when, as is sure to happen, some of the deeper villainies connected with quack advertisements come before him.

### The Metropolitan Asylums Board.

When an institution has done such good work as that which may properly be credited to the Metropolitan Asylums Board, one feels a certain hesitation in suggesting that the time has come for its abolition. Yet when one considers the very varied duties which this body has had imposed upon it from time to time it is difficult to avoid the conclusion that an occasion like the present, when fresh arrangements are being proposed for the government of the metropolis, is a fitting opportunity for restoring to the various authorities the work which properly belongs to them. It seems sufficiently obvious that the new municipalities ought to have some say in the management of the isolation hospitals on which they have so largely to depend in their attempts to control outbreaks of infectious disease within their several areas, while even those who admit to the fullest the good work done by the Asylums Board in regard to ambulance and hospitals find it difficult enough to defend the association of this work with the other duties which the Board has to perform—duties having to do with education, lunacy, the training of sailors, and the management of defective and vicious children. The lunatics might well go to the county, and the children to the School Board, while in regard to the hospitals, if they remain under the control of a special board, that board surely should be a department of the combined sanitary authorities, including the County Council, and not, as at present is the case, be appointed exclusively by the Poor Law guardians and the Local Government Board. As for the financial side of the question, it seems impossible to defend a system according to which the expense of so important a branch of sanitary administration as the isolation of infectious disease falls entirely on the poor rates.

### Vaccination and Insurance.

The *British Medical Journal* is publishing the results of a very useful inquiry which has been made as to the manner in which the various insurance offices deal with the question of vaccination. It is quite clear that as time goes on, unless the vaccination of the country is considerably improved, there will be a considerably increased mortality from small-pox, and that in any insurance office which accepts proposals from unvaccinated persons those who have protected themselves by vaccination will have to pay an undue share to make up the loss caused by the mortality among those who have not taken this precaution. This is likely to become a very important factor in the selection of an office. It is one of the axioms that an office which accepts none but good lives can offer the best terms, and that one which accepts risky lives must make up for its greater losses, either by demanding higher premiums, or by giving smaller bonuses, or by running greater risks in its investments. The office to avoid by the ordinary healthy man is, then, the one that accepts risky lives; and the unvaccinated man is undoubtedly a risky life. To ask an increased premium from those going to Africa because they may happen to catch malaria, and then to accept men who any day may develop small-pox even at home would be absurd, and we are glad to see that many of the insurance offices look at the matter in this light, and either refuse to have anything to do with unvaccinated people, or only accept them on special terms. Those which do not adopt this common sense course are clearly offices to avoid.

